# Synthesis of New Complex Ferrite Li_0.5_MnFe_1.5_O_4:_ Chemical–Physical and Electrophysical Research

**DOI:** 10.3390/ma17153754

**Published:** 2024-07-30

**Authors:** Mukhametkali Mataev, Altynai Madiyarova, Gennady Patrin, Moldir Abdraimova, Marzhan Nurbekova, Zhadyra Durmenbayeva

**Affiliations:** 1 Department of Chemistry, Faculty of Natural Sciences, Kazakh State Women’s Teacher Training University, Almaty 050000, Kazakhstan; mataev_06@mail.ru (M.M.); abdraimova87@mail.ru (M.A.); nurbekova.0@qyzpu.edu.kz (M.N.); durmenbayeva@mail.ru (Z.D.); 2 Department of General Physics, Siberian Federal University, Krasnoyarsk 660041, Russia; patrin@iph.krasn.ru

**Keywords:** sol–gel method, ferrites, X-ray, pycnometric density, elementary cell parameters, scanning electron microscope

## Abstract

In this article, the sol–gel method was used as a synthesis method, which shows the physicochemical nature of the synthesis of a new complex material, ferrite Li_0.5_MnFe_1.5_O_4_. The structure and composition of the synthesized ferrite were determined by X-ray phase analysis. According to analysis indicators, it was found that our compound is a single-phase, spinel-structured, and syngony-cubic type of compound. The microstructure of the compound and the quantitative composition of the elements contained within it were analyzed under a scanning electron microscope (SEM). Under a scanning electron microscope, microsystems were taken from different parts of Li_0.5_MnFe_1.5_O_4_-type crystallite; the elemental composition of crystals was analyzed; and the general type of surface layer of complex ferrite was shown. As a result, given the fact that the compound consists of a single phase, the clarity of its construction was determined by the topography and chemical composition of the compound. As a result, it was found that the newly synthesized complex ferrites correspond to the formula Li_0.5_MnFe_1.5_O_4_. The particles of the formed compounds have a large size (between 50.0 μm or 20.0 μm and 10.0 μm). Electrophysical measurements were carried out on an LCR-800 unit at intervals of 293–483 K and at frequencies of 1.5 and 10 kHz. An increase in frequency to 10 kHz led to a decrease in the value ε in the range of the studied temperature (293–483 K).

## 1. Introduction

Research in nanotechnology is a rapidly developing field at the forefront of physics and continues to be of particular interest to the scientific community [1]. Multifunctional materials, in particular, are studied for their effective properties combined in one smart compound [2,3]. A wide range of intelligent materials has been presented, and among them, significant attention has been paid to ferrites, which are considered promoter hosts due to their very prominent features [4,5].

Important results in this category of ceramics generally show excellent physicochemical properties, such as environmental mental health [6], chemical stability [7], high electrical resistance [8], and low price availability [9]. It is worth noting that in order for spinel ferrites to be useful for potential technological applications, a promising road was laid. Some prominent review articles indicate that the use of spinel ferrites for biomedical applications may be unavoidable due to their high biocompatibility [10] and low toxicity [6]. They have also been shown to have special properties for magnetic hyperthermia and magnetic resonance imaging [11,12]. Similarly, many discoveries have confirmed the development of this type of material as a suitable approach for ferrofluidic technology [13], wastewater treatment, catalysts [14], selective sun absorbers [15], and medical instruments [16].

With impressive properties such as conductivity, magnetic behavior, and high-temperature magnetic transition, mixed ferrites are soft ferrimagnetic compounds that show that they already exist in the field of technology. These materials are important due to their careless magnetization in room conditions [17,18].

The widespread use of spinel ferrites is due to their low price, easy preparation, and abundant use in technological and industrial applications [19]. The technological and industrial applications of these magnetic materials are due to their excellent electrical and magnetic properties, which leads to their use in magnetic memory devices, transformers, high-frequency applications, and electronic devices, such as mobile phones, camcorders, laptops, and computers [20].

Many researchers have studied the structural, electrical, and magnetic properties of various Li-based ferrites, such as Li-Mn [21], Li-Ni [22], Li-Co [23], Li-Mg [24], Li-Cr [25], Li-Zn [26], and Li-Ti [27], which are manufactured by a ceramic method. However, ferrite prepared by the ceramic method involves the synthesis of high calcination temperatures to complete the solid-state reaction between the constituent oxides or carbonates, and the particles are obtained in significantly larger and uneven sizes. This leads to the formation of voids, and then low-density ferrites, when uneven particles are compacted. To overcome these difficulties, the sol–gel method is the preferred method for synthesizing nanoferrite on a volumetric scale by obtaining homogeneous particles, and the most convenient method for synthesizing nanoparticles. This is because of the simplicity of this method; the low-cost precursors; the fact that the annealing process is carried out in a short time; good control of the amount of crystallite; and other properties of materials [28].

Nanoscale manganese ferrites exhibit interesting structural and magnetic properties. They have a wide range of applications, from basic research to industrial applications. In this work, the composition of ferrite nanoparticles is studied.

## 2. Materials and Methods

The sol–gel method was used as an effective way to synthesize a new mixed ferrite of a complex mixed composition. In order to determine the composition of a new complex mixed ferrite obtained by the sol–gel method, an X-ray phase study was carried out, and in order to conduct quantitative and qualitative analysis, an examination under a scanning electron microscope and a study of electrophysical properties (dielectric conductivity and electrical resistance) were carried out.

As a primary raw material, distilled water of a manganese (III) oxide (“chemical pure”) brand, iron (III) oxide (“chemical pure”) brand, and lithium conbanate (“chemical pure”) brand were used. Trihydric alcohol–glycerin and citric acid were used as gel-forming reagents. The raw materials obtained by stoichiometric calculation were weighed on an analytical balance with a maximum accuracy of 0.0001 g, mixed, ground in an agate sieve, placed in an Alund crucible, and fired in a muffle furnace at a temperature of 1100 °C.

The resulting dried gel was fired in a muffle oven for 10 h at a temperature of 600 °C until a black powder was obtained. Finally, the resulting powder was fired in stages for 7 h at a temperature of 700–1100 °C in the air [29].

The pycnometric density of ferrites was determined by a previously described method [30]. Toluene and distilled water were used as indifferent liquids. The density of composite materials was measured five times, and the average values were calculated.

## 3. Results and Discussion

### 3.1. X-Ray Analysis

X-ray analysis was carried out at the Kazakh National Women’s Pedagogical University on a diffractometer Miniflex/600 (Rigaku. Almaty, Kazakstan). Analysis using a sika beam (U = 30 KV, J = 10 MA, rotation speed 1000 pulses per second, time constant t = 5 s, 2θ, with an angle interval between 5 and 900) was carried out on a Miniflex 600 RIGAKU, and filtered by a filter.

**Figure 1:**
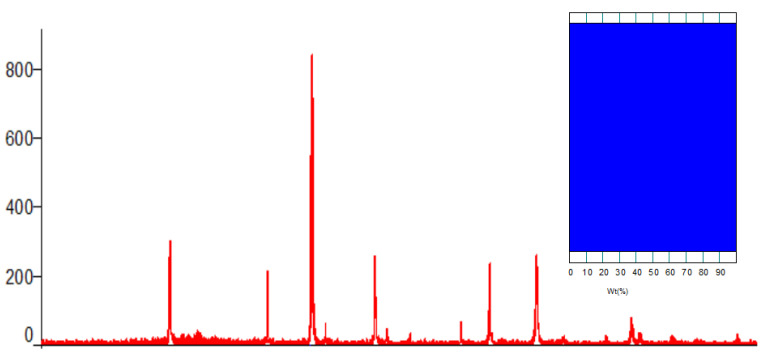
X-ray diffractogram of the complex ferrite Li_0.5_MnFe_1.5_O_4_. Insert: phase ratio diagram.

During the X-ray observation of the synthesized mixed complex ferrite, it was observed that the amorphous state of the samples decreased; the crystallization process was complete; and the kinetics of the sol–gel reaction were low. In addition, it is proved from the diffractogram shown in Figure 1 below that the samples changed from an amorphous state to a fully polycrystalline state, and the independent phase was completely formed.

Below are the results of the Rietveld method of indexing the diffractogram of the complex ferrite studied by X-ray analysis.

According to Scherrer’s formula, the average size of crystallites can be written as follows:

(1) $$d=\frac{K\lambda }{\beta \cos\theta }$$

where

*d*—average size of crystals;

*K*—dimensionless particle shape coefficient (Scherrer constant);

*λ*—wavelength of X-ray radiation;

*β*—half-height reflex width (2θ in radians and units);

*θ*—diffraction angle (Bragg angle).

A new complex mixed ferrite synthesized by the X-ray phase analysis method has the unit number Z = 8. Li_0.5_MnFe_1.5_O_4_ is crystallized in a cubic wall-centered cell, and the space group is $Fd\bar{3}m.$ Cell parameters: a = 8.3677, b = 8.3677, and c = 8.3677. The average size of crystallites according to Scherrer’s formula through the X-ray wavelength of ferrite is 40.6 µm, and the correctness of the results of the X-ray studies was confirmed by the coincidence of the values of X-ray and pycnometric densities.

### 3.2. Scanning Electron Microscope Observations

The spectrum of the distribution of the element was studied by a scanning electron microscope (Sam) (Application Note team, Brooker, Berlin, Germany) in order to conduct quantitative and qualitative analysis, and study the percentage content of elements.

A scanning electron microscope (SEM) is designed to obtain a magnified image of an object by scanning it with an electron beam directed at it and recording the signal generated by the interaction of electrons with a detector. The small diameter of the probe, even at low accelerating voltages and high currents, allows for elemental analysis of samples with dimensions of the analyzed area of several tens of nanometers. The beam current detector is located on the microscope column below the aperture of the objective lens, so that the beam current can be monitored at any time during the analysis.

**Figure 2:**
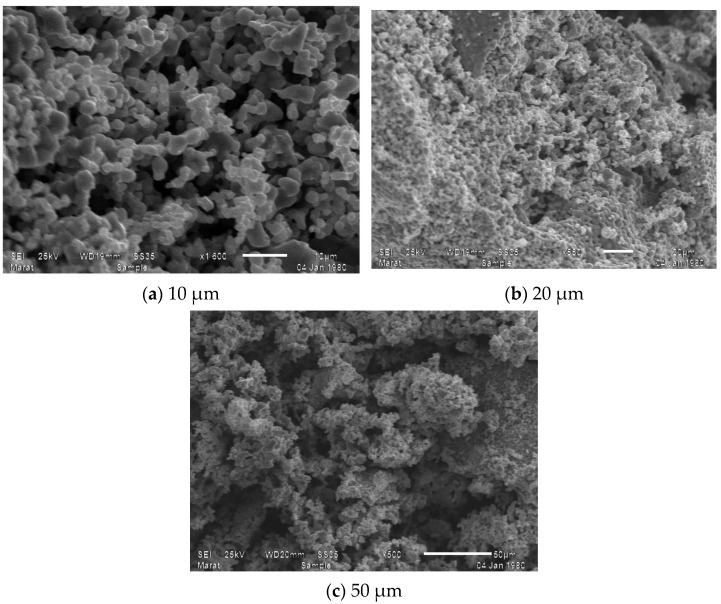
Image of the new mixed complex ferrite Li_0.5_MnFe_1.5_O_4_ measured with three different micrometer accuracies: (**a**) 10 μm; (**b**) 20 μm; (**c**) 50 μm.

To study the morphology of the surface layer of samples of new complex mixed ferrite synthesized by the sol–gel method, an image of electric diffraction was studied using an electron microscope scanning luminous microstructures. Electron monographs of the compound taken in an imaging electron microscope are given in Figure 2a–c.

**Figure 3:**
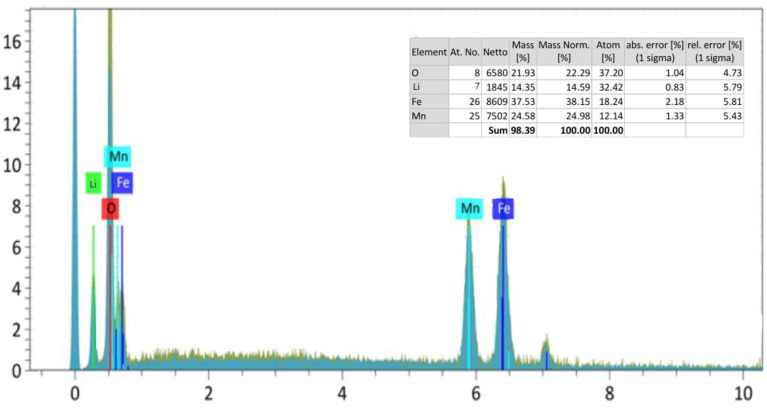
Spectrum samples of the Li_0.5_MnFe_1.5_O_4_ compound. The results of the element analysis are built-in.

The above images show the results of micrographs taken at magnifications of 10.0 μm, 20.0 μm, and 50.0 μm, and also show the general appearance of the complex ferrite surface layer. As a result, given that the compound consists of one phase, the clarity of its structure was determined by the topography and chemical composition of the compound. Figure 3 shows the spectrum patterns of the newly synthesized mixed complex ferrite Li_0.5_MnFe_1.5_O_4_ and the elemental analysis results of the Li_0.5_MnFe_1.5_O_4_ compound.

Based on the distribution map of elements, on the basis of solving the nature of crystallization, the chemical composition with microstructure and distribution zones of chromium, iron, lithium, and oxygen atoms were studied. As a result of the numerical elemental composition study, it can be concluded that iron, chromium, lithium metals, oxygen, and carbon atoms are distributed in the 10.0 μm regions (Figure 2a). With an imaging electron microscope, it is possible to obtain nanoscale measurements of solids in powder form.

The spectrum of the distribution of the element was studied using a scanning electron microscope in order to conduct quantitative and qualitative analysis, and study the percentage content of elments. The spectrum samples of the synthesized new mixed complex ferrite and the results of the elemental analysis are shown in Figure 3.

### 3.3. Electrophysical Research

This was carried out according to methods of measuring electrophysical properties [31,32].

The study of electrophysical properties (dielectric constant and electrical resistance) was carried out by measuring the electrical capacitance of samples in the serial device LCR-800 (Taiwan) in thermostatic mode at an operating frequency of 1 kHz in continuous dry air, with holding time at each set temperature.

Samples parallel to the plane in the form of discs with a diameter of 10 mm and a thickness of 2–6 mm were pre-prepared with a binder mixture (1.5%). Pressing was carried out at a pressure of 20 kg/cm^2^. The resulting discs were fired in the laboratory furnace “SNOL” at a temperature of 400 °C for 6 h. Then, they were carefully leveled on both sides.

The dielectric constant was determined from the electrical capacitance of the sample at certain values of the sample thickness and electrode surface. A Sawyer–Tower circuit was used to obtain the relationship between the electric induction d and the electric field strength E. Visual control of D (hysteresis loop e) was carried out on a C1-83 oscilloscope with a voltage divider consisting of a resistance of 6 Mom and 700 Kohm and a reference capacitor of 0.15 µF. The frequency of the generator was 300 Hz. In all temperature studies, samples were placed in a furnace; the temperature was measured with a chromium–aluminum thermocouple connected to a voltmeter B2-34 with an error of 0.1 MV. The temperature change rate was 5 K/min. The value of the dielectric constant at each temperature was determined by the following formula:

(2) $$\varepsilon =\frac{C}{C_{0}}$$

where $C_{0}=\frac{\varepsilon_{0}\cdot S}{d}$ is the capacitance of the capacitor without the test substance (air).

Calculation of the width of the forbidden zone (Δ*E*) of the substance under investigation was determined by the following formula:

(3) $$\Delta E=\frac{2kT_{1}T_{2}}{0.43(T_{2}-T_{1})}\mathrm{lg}\frac{R_{1}}{R_{2}},$$

where *k* is the Boltzmann constant equal to 8.6173303 ×·10^−5^ eB·K^−1^; *R*_1_ is the resistance at *T*_1_; and *R*_2_ is the resistance at *T*_2_.

To ensure the reliability of the data obtained, the dielectric constant of a standard substance, barium titanate BaTiO_3_, was measured at frequencies equal to 1 kHz, 5 kHz, and 10 kHz. Table 1 below shows the results of measurements of the electrophysical characteristics of BaTiO_3_.

As can be seen from the data in Table 2, the values of the dielectric constant 293 K at 1 kHz and 5 kHz correspond satisfactorily to its recommended value of 1400 ± 250. In addition, observed changes in the electrical conductivity of BaTiO_3_ at all frequencies (1 kHz, 5 kHz, and 10 kHz) at 383 K correspond to its transition from the perovskite cubic phase Pm3m to the tetragonal (polar) ferroelectric phase with the space group P4mm [33,34,35].

It should be noted that despite the decrease in the values of the dielectric constant of BaTiO_3_ at a frequency of 10 kHz, and at T equal to 293 K, 303 K, and 313 k, and all values of ε BaTiO_3_ at all three frequencies (1 kHz, 5 kHz, and 10 kHz) in the range of 313–483 K, there are values up to about 2150, indicating that this frequency change does not particularly affect the temperature dependence of the dielectric constant. BaTiO_3_ is in the range of 313–483 K.

**Figure 4:**
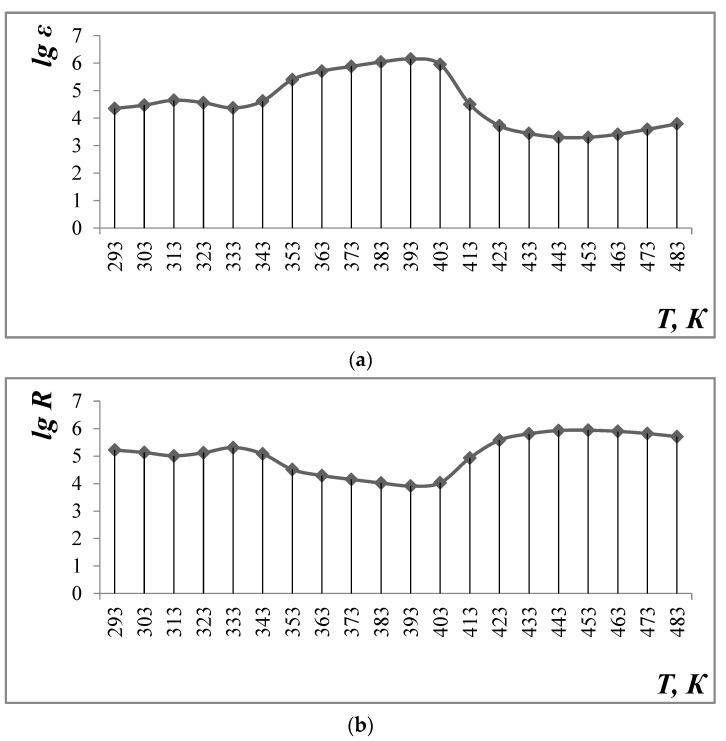
Dependence of dielectric constant (**a**) and electrical resistance (**b**) on temperature and frequency equal to 1 kHz.

Electrophysical measurements of Li_0.5_MnFe_1.5_O_4_ in the range of 293–483 K and frequencies equal to 1, 5, and 10 kHz were carried out at the LCR installation (Table 3, Figure 4).

The data in Table 3 and Figure 4 show that the value of the dielectric constant (ε) reaches a maximum value at 383 K, which is equal to 1.43·106 (1 kHz). Then, it decreases to 6140 at 483 K (frequency 1 kHz). Increasing the frequency to 5 and 10 kHz leads to a decrease in ε in the entire temperature range under study (293–483 K).

The study of the temperature dependence of electrical resistance shows the complex nature of conductivity: at ∆T = 293–313 K—semiconductor, at ∆T = 313–333 K—metallic, at 333–393 K—semiconductor, at ∆T = 393–453 K—metallic, and at ∆T = 453–483 K—again, semiconductor.

The band spacing of 2 materials AT ∆T = 293–313 K (Table 4) and 333–393 K (Table 5) is equal to 1.91 and 1.19 EV (narrowband semiconductor), respectively, and AT ∆T = 453–483 K (Table 6) is equal to 3.04 EV (broadband semiconductor). 

## 4. Conclusions

Summing up the results of the study, a new mixed complex ferrite with the composition Li_0.5_MnFe_1.5_O_4_ was synthesized for the first time by the sol–gel method. In order to determine the composition of the resulting new complex mixed ferrite, an X-ray phase study was carried out, and in order to conduct quantitative and qualitative analysis, an examination under a scanning electron microscope and a study of electrophysical properties (dielectric conductivity and electrical resistance) were carried out.

For the first time, syngony types and parameters of elementary cells of a complex mixed ferrite synthesized by the method of X-ray phase analysis were determined. Li_0.5_MnFe_1.5_O_4_ (cubic, a = 8.3677(9), B = 8.3677(9), C = 8.3677(9) Å, Z = 8, denX-ray = 4.678 g/cm^3^, denPycno = 4.675 g/cm^3^); through the X-ray wavelength of ferrite, the average size of crystallites according to Scherrer’s formula is 40.6 µm. The results of radiographic research showed that the synthesized compound is polycrystalline. The accuracy of crystallochemical data is evidenced by a satisfactory correspondence of X-ray and pycnometric densities.

Through a scanning electron microscope, microsystems were taken from different parts of Li_0.5_MnFe_1.5_O_4_-type crystallite; the elemental composition of crystals was analyzed; and the general type of surface layer of complex ferrite was shown. As a result, given the fact that the compound consists of a single phase, the clarity of its construction was determined by the topography and chemical composition of the compound. As a result, it was found that the newly synthesized complex ferrites correspond to the formula Li_0.5_MnFe_1.5_O_4_. The particles of the formed compounds have a large size (between 50.0 µm or 20.0 µm and 10.0 µm). The results of the elemental analysis were presented in the form of a table.

Electrophysical measurements of Li_0.5_MnFe_1.5_O_4_ on the LCR-800 unit were carried out at intervals of 293–483 K and frequencies of 1.5 and 10 kHz.

Given the fact that 293 K is equal to a relatively high value of 2.69 × 105 at 1 kHz, the maximum value of 383 K reached 1.43 × 106. Next, it was reduced to 6140 at 483 K (frequency of 1 kHz). An increase in frequency to 5 and 10 kHz led to a decrease in the entire test temperature interval (293–483 K).

The study of the dependence of electrical resistance on temperature shows the complex nature of conductivity: t = 293–313 K—semiconductor, t = 313–333 K—metal, 333–393 K—semiconductor, t = 393–453 K—metal, and T = 453–483 K—semiconductor again.

In the future, I will study the temperature dependence and magnetic sensitivity of the magnetization of synthesized complex ferrite in various magnetic fields.

## Figures and Tables

**Table 1:** Symmetry type and unit cell parameters of Li_0.5_MnFe_1.5_O_4._

Sample	Li_0.5_MnFe_1.5_O_4_
Space group	$F\bar{d}3m$, cubic side centered
Z Parameter cell (Å) a= b= c= V(A^3) α β γ Average size of crystallites according to Scherrer’s formula X-ray density (g/cm^3^) Pycnometric density (g/cm^3^) *	8 8.3677(9) 8.3677(9) 8.3677(9) 585.90(11) 90 90 90 40.6 μm 4.678 4.675

* Calculated manually by pycnometer.

**Table 2:** Dependence of electrical resistance (R), electrical capacity (C), and dielectric constant (ε) on BaTiO_3_ temperature.

T, K	C, nF	R, O_M_	ε	lgε	lgR
Measurement frequency at 1 kHz					
293 303 313 323 333 343 353 363 373 383 393 403 413 423 433 443 453 463 473 483	0.27278 0.27426 0.27715 0.28125 0.28772 0.29313 0.29916 0.30751 0.31202 0.31702 0.32255 0.32967 0.3423 0.35119 0.36668 0.38018 0.39802 0.4169 0.43147 0.45456	13,400 13,270 12,910 12,560 11,890 11,210 10,290 9383 8831 9061 8814 7881 7098 6902 6153 6317 6010 5584 5149 4656	1296 1303 1316 1336 1367 1392 1421 1461 1482 1506 1532 1566 1626 1668 1742 1806 1891 1980 2050 2159	3.11 3.11 3.12 3.13 3.14 3.14 3.15 3.16 3.17 3.18 3.19 3.19 3.21 3.22 3.24 3.26 3.28 3.30 3.31 3.33	4.13 4.12 4.11 4.10 4.08 4.05 4.01 3.97 3.95 3.96 3.95 3.90 3.85 3.84 3.79 3.80 3.78 3.75 3.71 3.67
Measurement frequency at 5 kHz					
293 303 313 323 333 343 353 363 373 383 393 403 413 423 433 443 453 463 473 483	0.25678 0.2683 0.2775 0.28638 0.29667 0.30226 0.30787 0.31283 0.31843 0.32148 0.32578 0.32976 0.33303 0.33948 0.35613 0.3713 0.3925 0.41682 0.44245	29,630 21,650 13,080 5236 4301 4733 3296 2966 2805 2529 2669 3172 4434 6377 9644 11,520 10,430 8021 5978 3799	1220 1274 1318 1360 1389 1409 1436 1462 1486 1513 1527 1547 1566 1582 1613 1692 1764 1864 1980 2102	3.09 3.11 3.12 3.13 3.14 3.15 3.16 3.17 3.17 3.18 3.18 3.19 3.19 3.20 3.21 3.23 3.25 3.27 3.30 3.32	4.47 4.34 4.12 3.72 3.63 3.68 3.52 3.47 3.45 3.40 3.43 3.50 3.65 3.80 3.98 4.06 4.02 3.90 3.78 3.58
Measurement frequency at 10 kHz					
293 303 313 323 333 343 353 363 373 383 393 403 413 423 433 443 453 463 473 483	0.11814 0.18494 0.22927 0.25954 0.27501 0.28531 0.29302 0.29988 0.30652 0.31215 0.31667 0.32294 0.32779 0.33406 0.34256 0.35658 0.378 0.39475 0.41687 0.44203	152,300 70,790 32,200 11,870 4842 3312 2689 2257 1946 1689 1737 3130 5945 8231 8805 8052 5967 4604 3343 2353	561 878 1089 1233 1306 1355 1392 1424 1456 1483 1504 1534 1557 1587 1627 1694 1796 1875 1980 2100	2.75 2.94 3.04 3.09 3.12 3.13 3.14 3.15 3.16 3.17 3.18 3.19 3.19 3.20 3.21 3.23 3.25 3.27 3.30 3.32	5.18 4.85 4.51 4.07 3.69 3.52 3.43 3.35 3.29 3.23 3.24 3.50 3.77 3.92 3.94 3.91 3.78 3.66 3.52 3.37

**Table 3:** Dependence of electrical capacity (C), electrical resistance (R), and dielectric constant (ε) of material 2 on temperature and frequency.

T, K	C, nF	R, O_M_	ε	lgε	lgR
Measurement frequency at 1 kHz					
293 303 313 323 333 343 353 363 373 383 393 403 413 423 433 443 453 463 473 483	7.7977 10.302 15.68 12.489 8.1684 14.516 86.933 176.65 265.14 378.56 495.27 312.87 11.039 1.8105 0.95809 0.68585 0.70009 0.89737 1.3483 2.1329	165,700 135,800 101,700 131,900 203,600 121,200 32,510 19,300 14,110 10,520 8184 10,720 85,500 379,400 649,900 843,600 874,900 796,100 666,100 511,600	22,448 29,658 45,140 35,954 23,515 41,789 250,266 508,547 763,295 1,089,813 1,425,802 900,702 31,779 5212 2758 1974 2015 2583 3882 6140	4.35 4.47 4.65 4.56 4.37 4.62 5.40 5.71 5.88 6.04 6.15 5.95 4.50 3.72 3.44 3.30 3.30 3.41 3.59 3.79	5.22 5.13 5.01 5.12 5.31 5.08 4.51 4.29 4.15 4.02 3.91 4.03 4.93 5.58 5.81 5.93 5.94 5.90 5.82 5.71
Measurement frequency at 5 kHz					
293 303 313 323 333 343 353 363 373 383 393 403 413 423 433 443 453 463 473 483	1.3926 1.8886 2.728 1.517 0.9064 2.5719 15.47 31.031 45.909 64.608 82.944 40.501 1.7403 0.28075 0.13233 0.09429 0.09084 0.10483 0.13985 0.19866	140,100 115,400 89,690 128,100 184,900 93,070 29,090 17,900 13,060 9799 7702 11,420 79,660 293,500 455,500 533,500 555,900 536,400 477,500 401,900	4009 5437 7853 4367 2609 7404 44,536 89,333 132,165 185,996 238,782 116,596 5010 808 381 271 262 302 403 572	3.60 3.74 3.90 3.64 3.42 3.87 4.65 4.95 5.12 5.27 5.38 5.07 3.70 2.91 2.58 2.43 2.42 2.48 2.60 2.76	5.15 5.06 4.95 5.11 5.27 4.97 4.46 4.25 4.12 3.99 3.89 4.06 4.90 5.47 5.66 5.73 5.74 5.73 5.68 5.60
Measurement frequency at 10 kHz					
293 303 313 323 333 343 353 363 373 383 393 403 413 423 433 443 453 463 473 483	0.62512 0.85581 1.188 0.50394 0.3238 1.2082 7.2162 14.584 21.676 30.192 39.355 14.483 0.64942 0.12128 0.06145 0.04865 0.04694 0.05137 0.06369 0.08415	126,300 103,700 83,510 127,500 169,200 76,460 26,640 16,680 12,440 9304 7426 12,940 83,960 243,700 329,700 351,900 362,500 360,400 339,200 306,100	1800 2464 3420 1451 932 3478 20,774 41,985 62,402 86,918 113,297 41,694 1870 349 177 140 135 148 183 242	3.26 3.39 3.53 3.16 2.97 3.54 4.32 4.62 4.80 4.94 5.05 4.62 3.27 2.54 2.25 2.15 2.13 2.17 2.26 2.38	5.10 5.02 4.92 5.11 5.23 4.88 4.43 4.22 4.09 3.97 3.87 4.11 4.92 5.39 5.52 5.55 5.56 5.56 5.53 5.49

**Table 4:** Calculation of the width of the forbidden zone (∆E) in the range 293–313 K.

T, K	lg R
293	5.22
313	5.01
$\Delta E=\frac{2\times 0.000086173\times 293\times 313}{0.43(313-293)}\mathrm{lg}\frac{5.22}{5.01}=1.91эB$	

**Table 5:** Calculation of the width of the forbidden zone (∆E) in the range 333–393 K.

T, K	lg R
333	5.31
393	3.91
$\Delta E=\frac{2\times 0.000086173\times 333\times 393}{0.43(393-333)}\mathrm{lg}\frac{5.31}{3.91}=1.19эB$	

**Table 6:** Calculation of the width of the forbidden zone (∆E) in the range 453–483 K.

T, K	lg R
453	5.94
483	5.71
$\Delta E=\frac{2\times 0.000086173\times 453\times 483}{0.43(483-453)}\mathrm{lg}\frac{5.94}{5.71}=3.04\;эB$	

## Data Availability

Data are contained within the article.
